# Polymorphisms in the Non-Muscle Myosin Heavy Chain Gene (MYH9) Are Associated with Lower Glomerular Filtration Rate in Mixed Ancestry Diabetic Subjects from South Africa

**DOI:** 10.1371/journal.pone.0052529

**Published:** 2012-12-20

**Authors:** Tandi Edith Matsha, Katya Masconi, Yandiswa Yolanda Yako, Mogamat Shafick Hassan, Muiriri Macharia, Rajiv Timothy Erasmus, Andre Pascal Kengne

**Affiliations:** 1 Department of Biomedical Sciences, Faculty of Health and Wellness Science, Cape Peninsula University of Technology, Cape Town, South Africa; 2 Division of Chemical Pathology, Faculty of Health Sciences, National Health Laboratory Service and University of Stellenbosch, Cape Town, South Africa; 3 Department of Nursing and Radiography, Faculty of Health and Wellness Science, Cape Peninsula University of Technology, Cape Town, South Africa; 4 NCRP for Cardiovascular and Metabolic Diseases, South African Medical Research Council & University of Cape Town, Cape Town, South Africa; University of Louisville, United States of America

## Abstract

**Objective:**

Though single nucleotide polymorphisms (SNPs) in the non-muscle myosin gene (MYH9) have been reported to explain most of the excess risk of nondiabetic chronic kidney disease (CKD), in African-Americans, some studies have also shown associations with diabetic end-stage renal disease. We investigated the association of MYH9 SNPs with renal traits in a mixed-ancestry South African population prone to diabetes.

**Research Design and Methods:**

Three SNPs known to be associated with CKD (rs4821480, rs5756152 and rs12107) were genotyped using Taqman assay in 716 adults (198 with diabetes) from the Bellville-South community, Cape Town. Glomerular filtration rate was estimated (eGFR) and urinary albumin/creatinine ratio (ACR) assessed. Multivariable regressions were used to relate the SNPs with renal traits.

**Results:**

Mean age was 53.6 years, with the expected differences observed in characteristics by diabetic status. Significant associations were found between rs575152 and serum creatinine, and eGFR in the total population, and in diabetic participants (all p≤0.003), but not in non-diabetics (all p≥0.16), with significant interactions by diabetes status (interaction-p≤0.009). The association with ACR was borderline in diabetic participants (p = 0.05) and non-significant in non-diabetics (p = 0.85), with significant interaction (interaction p = 0.02). rs12107 was associated with fasting-, 2-hour glucose and HbA1c in diabetic participants only (interaction-p≤0.003), but not with renal traits.

**Conclusion:**

MYH9 SNPs were associated with renal traits only in diabetic participants in this population. Our findings and other studies suggest that MYH9 may have a broader genetic risk effect on kidney diseases.

## Introduction

The characterization of the associations between chronic kidney diseases (CKD) and polymorphisms on chromosome 22q12, namely MYH9 and APOL1 was an immense breakthrough for population-based genetic studies of CKD [1], [2]. The MYH9 gene encodes for the non-muscle myosin IIA heavy chain, a subunit of myosin IIA protein [3]. This protein is involved in several important functions, including cytokinesis, cell motility, maintenance of cell shape and specialized functions such as secretion. Autosomal-dominant mutations in MYH9 are linked to the development of the giant platelet syndrome disorders, which in turn are also associated with susceptibility of progression to renal failure of glomerular origin [4].

African Americans have been reported to show a disproportionate risk for specific forms of CKD such as focal segmental glomerulosclerosis (FSGS) [5], diabetic nephropathy [6], hypertensive nephrosclerosis [7], lupus nephritis [8], and HIV associated nephropathy (HIVAN) [9]. In 2008 two studies identified single nucleotide polymorphisms (SNPs) in the MYH9 gene which conferred most of the increased risk for nondiabetic kidney disease in African Americans [1], [2]. The MYH9 SNPs were clustered into groups according to linkage disequilibrium blocks: extended or E-1 haplotype (rs4821480, rs2032487, and rs4821481, rs3752462) [1], [2], S-1 (rs5750248, rs2413396, rs5750250), F-1 (rs16996674, rs16996677, rs11912763) [10] and L1 (rs12107, rs7078, rs735853, rs5756129) [11]. Furthermore, some of the SNP’s (rs2032387, rs1699667, rs821480, rs575152, rs12107) exhibited independent evidence for association with non-diabetic end-stage renal disease (ESRD) [11]. Nevertheless, some reports also demonstrated an association between MYH9 gene polymorphisms and diabetic ESRD in African-Americans [12], [13].

Polymorphisms in the MYH9 gene in relation with kidney disease have not yet been investigated in mixed ancestry African populations. Thus, our aim was to explore the association between MYH9 SNPs and renal traits in the mixed ancestry population of South Africa which has previously been shown to have a high prevalence of diabetes [14].

## Research Design and Methods

### Ethical Considerations

The study was approved by the Cape Peninsula University of Technology, Faculty of Health and Wellness Sciences ethics committee (Reference Number: CPUT/HW-REC 2008/002 and CPUT/HW-REC 2010/H017). The study was conducted according to the Code of Ethics of the World Medical Association (Declaration of Helsinki). All participants signed written informed consent after all the procedures had been fully explained in the language of their choice. In addition, permission was also sought from other relevant authorities such as the city and community authorities. These authorities granted permission to operate in the community and also to make use of designated places such as community halls or nearby schools for data and samples collection.

### Study Setting and Population

Participants were members of a cohort study conducted in Bellville-South, Cape Town, a mixed ancestry Township formed in the late 1950s. A detailed description of the survey design and procedures has been previously described [14], [15] Briefly, eligible participants, selected from 1000 households through a multistage random sampling, were invited to take part in a community based survey between January 2008 and March 2009, with data collection through standardized procedures.

### Clinical Data

All consenting participants received a standardized interview and physical examination during which blood pressure was measured according to World Health Organisation (WHO) guidelines [16] using a semi-automatic digital blood pressure monitor (Rossmax PA, USA) on the right arm in sitting position. Other clinical measurements included the body weight, height, waist and hip circumferences. Weight (to the nearest 0.1 kg) was determined with a Sunbeam EB710 digital bathroom scale, which was calibrated and standardized using a weight of known mass. Weight measurements were recorded with each subject wearing light clothing, without shoes and socks. Waist circumference was measured using a non-elastic tape at the level of the narrowest part of the torso, as seen from the anterior view. All anthropometric measurements were performed three times and their average used for analysis. Participants with no history of doctor diagnosed diabetes mellitus underwent a 75 g oral glucose tolerance test (OGTT) as recommended by the WHO [17].

### Laboratory Measurements

Blood samples were collected after an overnight fast and processed for further biochemical analysis. Plasma glucose was measured by enzymatic hexokinase method (Cobas 6000, Roche Diagnostics, Germany) and glycated haemoglobin (HbA1c) by turbidimetric inhibition immunoassay (Cobas 6000, Roche Diagnostics, Germany). This method is National Glycohaemoglobin Standardisation Programme (NGSP) certified according to Roche Diagnostics. Creatinine measurements were done using the standardized creatinine assay (Cobas 6000, Roche Diagnostics, Germany) and urine albumin by the immunoturbidimetric assay (Cobas 6000, Roche Diagnostics, Germany).

### Definitions and Calculations

Diabetes status was based on a history of doctor-diagnosis, a fasting plasma glucose > = 7.26 mmol/l and/or a 2-hour post-OGTT plasma glucose >11.1 mmol/l. Hypertension was based on a history of doctor diagnosed hypertension and/or receiving medications for hypertension or average systolic blood pressure ≥140 mmHg and/or average diastolic blood pressure ≥90 mmHg. Urinary albumin excretion was quantified in term of urinary albumin/creatinine ratio (ACR) and participants ranked according to ACR levels as: normoalbuminuria (ACR<3.4 mg/mmol), microalbuminuria (3.4≤ACR<33.9 mg/mmol) and macroalbuminuria (ACR≥33.9 mg/mmol). Kidney function was assessed through estimated glomerular filtration rate (eGFR) for which both the 4-variable Modification of Diet in Renal Disease (MDRD) equation [18], [19] applicable to standardised serum creatinine values, and the Chronic Kidney Disease Epidemiology Collaboration (CKD-EPI) equation [20] were used, assuming a black ethnicity for all participants. An eGFR<60 ml/min was used to defined chronic kidney disease (or CKD stage 3–5).

### SNP Selection and Genotyping

There is no published data on MYH9 SNPs and haplotype variations in mixed ancestry population from South Africa. We therefore selected three SNPs that have been shown to exhibit independent evidence for association with CKD (rs4821480, rs575152 and rs12107) in prior studies [11]. The SNPs were genotyped using Taqman genotyping assay (Applied Biosystems, USA) on a BioRad Optica (Biorad, USA) and confirmed by sequencing.

### Statistical Analysis

Of the 946 participants who took part in the survey, 941 consented for genetic studies. Among the latter, 226 were excluded for missing data on the genetic or renal trait variables. Therefore, 716 had valid data for the current analyses. General characteristics of the study group are summarized as count and percentage for dichotomous traits, mean and standard deviation (SD) or median and 25^th^–75^th^ percentiles for quantitative traits. Traits were log-transformed to approximate normality, where necessary, prior to analysis. SNPs were tested for departure from Hardy-Weindberg Equilibrium (HWE) expectation via a chi square goodness of fit test. Linkage disequilibrium (LD) was estimated using the D’ statistic. Linear regression models were used for the analysis of quantitative traits and logistic regression models for dichotomous traits, always assuming additive models for the SNPs. Using linear and logistic models enabled us to adjust all analyses for known confounders as specified everywhere in the results. We investigated the additive allelic association of each SNP with each trait, overall and according to diabetes status, and tested for heterogeneity by adding the interaction term of diabetes and each SNP to a model that contained the main effects of diabetes and the relevant SNP. Results corresponding to p-values below 5% are described as significant. We did not adjust for multiple testing. All analyses used the statistical package R (version 2.13.0 [2011–04–13], The R Foundation for statistical computing, Vienna, Austria).

## Results

### Baseline Profile

Table 1 summarizes the clinical and biological profile of the 716 participants. One hundred and ninety eight participants (27.7%) had type 2 diabetes. Several expected differences according to diabetes status were apparent in the study sample. For instance, compared with nondiabetics, participants with diabetes were older (59.9 vs. 51.3 years, p<0.0001), had higher body mass index (31.7 vs. 29.1 kg/m2, p<0.0001), higher waist-to-hip ratio (0.92 vs. 0.87, p<0.0001), higher systolic blood pressure (129 vs. 121 mmHg, p<0.0001) and included more individuals with hypertension (65.1% vs. 34.0%, p<0.0001). Furthermore, they were more likely to have higher urinary albumin/creatinine ratio (median: 1.06 vs. 0.62, p<0.0001) and lower estimated glomerular filtration rates based on either the MDRD, or CKD-EPI formulae (both p≤0.01), Table 1.

**Table 1:** Baseline characteristics overall and by diabetes status.

Characteristics	No diabetes	Diabetes	P-value	Overall
N	518	198		716
Gender, men n (%)	113 (21.8)	45 (22.7)	0.79	158 (22.1)
Mean age, years (SD)	51.3 (15.1)	59.9 (12.4)	<0.0001	53.6 (14.9)
Mean BMI, kg/m^2^ (SD)	29.1 (7.3)	31.7 (7.0)	<0.0001	29.8 (7.3)
Mean WHR (SD)	0.87 (0.08)	0.92 (0.08)	<0.0001	0.88 (0.08)
Mean SBP, mmHg (SD)	121 (18)	129 (20)	<0.0001	123 (19)
Mean DBP, mmHg (SD)	74 (12)	76 (13)	0.09	75 (12)
Hypertension, yes (%)	176 (34.0)	129 (65.1)	<0.0001	305 (42.3)
Mean HbA1c, % (SD)	5.7 (0.4)	7.7 (1.9)	<0.0001	6.2 (1.4)
Mean FBG, mmol/l (SD)	5.2 (0.7)	9.8 (4.1)	<0.0001	6.5 (3.0)
Mean 2-h glucose, mmol/l (SD)	6.5 (1.6)	13.3 (4.8)	<0.0001	7.7 (3.4)
Median urine creatinine, mmol/l (25^th^–75^th^ percentiles)	8.1 (7.2–12.2)	6.6 (4.3–11.2)	0.02	7.8 (5.1–11.9)
Median urine Microalbumin, mg/l (25^th^–75^th^ percentiles)	3.6 (3.0–9.6)	7.8 (3.0–25.6)	<0.0001	4.3 (3.0–11.8)
Median ACR, mg/mmol (25^th^–75^th^ percentiles)	0.62 (0.37–1.21)	1.06 (0.59–3.21)	<0.0001	0.74 (0.41–1.54)
Normoalbuminuria (<3.4), n (%)	479 (92.5)	153 (77.3)	<0.0001	632 (88.3)
Microalbuminuria([3.4–33.9[), n (%)	38 (7.3)	31 (15.7)		69 (9.6)
Macroalbuminuria (> = 33.9), n (%)	1 (0.2)	14 (7.1)		15 (2.1)
Albuminuria (> = 3.4), n (%)	39 (7.5)	45 (22.7)	<0.0001	84 (11.7)
Median serum creatinine,µmol/l (25^th^–75^th^ percentiles)	81 (70–91)	85 (71–98)	0.04	81 (71–93)
Mean eGFR (MDRD), ml/min (SD)	87.6 (22.0)	82.1 (26.7)	0.01	86.1 (23.5)
eGFR (MDRD) <60, n (%)	38 (7.3)	30 (15.2)	0.001	68 (9.5)
Mean eGRF (CKD-EPI), ml/min (SD)	92.1 (23.4)	83.0 (25.6)	<0.0001	89.6 (24.4)
eGFR (CKD-EPI) <60, n (%)	36 (6.9)	31 (15.7)	0.0003	67 (9.4)

ACR, urinary albumin/creatinine ratio; CKD-EPI, Chronic Kidney Disease Epidemiology Collaboration; eGFR, estimated glomerular filtration rate; MDRD, Modification of Diet in Renal Disease; SD, standard deviation.

### SNP Distribution

SNP rs5756152 was in linkage disequilibrium (LD) with both rs4821480 (D′ = 0.682) and rs12107 (0. 169) in the overall cohort, and in participants with diabetes (D′ = 0.797 and 0.171) and those without diabetes (D′ = 0.636 and 0.168). SNP rs4821480 was in weak LD with rs12107 in all the groups: participants without diabetes (D′ = 0.147), participants with diabetes (D′ = 0.170), overall cohort (D′ = 0.089). All the SNPs were in HWE (all p>0.05) except rs4821480 in the overall cohort (p = 0.006) and in participants with diabetes (p = 0.037). Therefore, rs4821480 was removed from further analysis. The frequency distributions, both genotype and allele, did not differ significantly according to diabetes status. The A allele of rs575152, G allele of rs12107 and G allele of rs4821480 were the most frequent overall and in participants with and without diabetes (Table 2).

**Table 2:** Genotype distributions, minor allele frequencies, and unadjusted p-values for comparing genotype distributions according to diabetes status, additive allelic effects between diabetes groups.

	No diabetes	Diabetes	p-value	Overall
N	518	198		716
**rs5756152**				
A/A, n (%)	389 (75.1)	149 (75.2)	0.775	538 (75.1)
A/G, n (%)	120 (23.2)	44 (22.2)		164 (22.9)
G/G, n (%)	9 (1.7)	5 (2.5)		14 (1.9)
G, n (%)	129 (12.4)	49 (12.4)	>0.999	178 (12.4)
HWE (p-value)	>0.999	0.376		0.746
**rs4821480**				
G/G, n (%)	202 (39.0)	80 (40.4)	0.710	282 (39.4)
G/T, n (%)	226 (43.6)	80 (40.4)		306 (42.7)
T/T, n (%)	90 (17.4)	38 (19.2)		128 (17.9)
T, n (%)	316 (30.5)	118 (29.8)	0.847	434 (30.3)
HWE (p-value)	0.053	0.037		0.006
**rs12107**				
G/G, n (%)	286 (55.2)	112 (56.6)	0.930	398 (55.6)
G/A, n (%)	199 (38.4)	73 (36.9)		272 (38.0)
A/A, n (%)	33 (6.4)	13 (6.6)		46 (6.4)
A, n (%)	232 (22.4)	86 (21.7)	0.831	318 (22.2)
HWE (p-value)	0.908	0.850		>0.999

HWE, Hardy-Weindberg Equilibrium (HWE p-values are from exact tests).

### Association between SNPs within MYH9 and Renal Traits

The additive allelic effect of the SNPs on the traits differed significantly between participants with and without diabetes. The results are summarized Table 3. For each of the combinations of trait and SNP showing a difference in the overall cohort, the effect was significant only in those with type 2 diabetes where it was further magnified in stratified analyses. For instance, in participants with diabetes, with each G allele of rs5756152, serum creatinine decreased by 13% (95% confidence interval: 6.2–19.7%), and eGFR (MDRD) or eGFR (CKD-EPI) increased by 11.08 (5.21–16.96) and 10.01 (4.85–15.33) ml/min respectively, after adjustment for age, sex and urinary albumin excretion (log_e_). Equivalents figures in nondiabetics were 2.2% (−0.9 to 5.6%), 1.91 (−1.32 to 5.15) and 1.83 (−1.23 to 4.89), with significant interactions in all according to diabetes status (all interaction p≥0.009). rs12107 was not associated with serum creatinine and eGFR. Associations of the SNPs with urinary albumin excretion tended to be significant in participants with diabetes (all p≤0.10), but not in those without (all p≥0.74), with also a trend toward significant heterogeneity by diabetes status (all interaction p≤0.06), Table 3.

**Table 3:** Generalized linear regression models showing the effects of genes on kidney functions and other continuous predictors.

		Overall		diabetes		No diabetes		pinteraction
Allele	Phenotype	Effects size (95%CI)	p	Effects size (95%CI)	p	Effects size (95%CI)	p	
rs5756152 G	Serum creatinin (log_e_[µmol/l])	−5.1% (−8.2 to −2.1)	0.001	−13.0% (−19.7to −6.2)	0.0002	−2.3% (−5.6 to 0.9)	0.16	0.004
	eGFR (MDRD) [ml/min]	4.71 (1.79 to 7.65)	0.002	11.08 (5.21 to 16.96)	0.0003	1.91 (−1.32 to 5.15)	0.25	0.003
	eGFR (CKD-EPI) [ml/min]	4.04 (1.37 to 6.70)	0.003	10.01 (4.85 to 15.33)	0.0002	1.83 (−1.23 to 4.89)	0.24	0.009
	Fasting blood glucose (mmol/l)	0.37 (0.04 to 0.71)	0.030	0.91 (−0.25 to 2.07)	0.13	0.14 (0.01 to 0.27)	0.03	0.03
	2-hour blood glucose (mmol/l)	0.04 (−0.33 to 0.41)	0.82	0.86 (−1.15 to 2.87)	0.81	−0.11 (−0.38 to 0.15)	0.41	0.04
	HbA1c (%)	0.04 (−0.12 to 0.20)	0.63	0.29 (−0.27 to 0.85)	0.31	−0.08 (−0.15 to −0.01)	0.03	0.02
	Albumin/creatinine ratio (mg/mmol)	10.8% (−6.4 to 28.0)	0.22	43.6% (0.1 to 87.1)	0.05	−1.6% (−18.9 to 15.6)	0.85	0.02
	Systolic blood pressure (mmHg)	−1.35 (−3.99 to 1.29)	0.32	−0.63 (−6.27 to 5.02)	0.83	−1.76 (−4.73 to 1.22)	0.25	0.65
rs12107 A	Serum creatinin (log_e_[µmol/l])	0.05% (−2.4 to 2.5)	0.97	−0.2% (−5.7 to 5.2%)	0.93	0.4% (−2.2 to 2.9)	0.78	0.60
	eGFR (MDRD) [ml/min]	0.06 (−2.26 to 2.37)	0.96	−0.17 (−5.05 to 4.71)	0.94	−0.07 (−2.61 to 2.46)	0.95	0.84
	eGFR (CKD-EPI) [ml/min]	0.37 (−1.73 to 2.48)	0.73	0.68 (−3.55 to 4.90)	0.75	0.13 (−2.27 to 2.54)	0.91	0.62
	Fasting blood glucose (mmol/l)	−0.31 (−0.57 to −0.04)	0.022	−0.90 (−1.81to −0.004)	0.05	−0.07 (−0.17 to 0.03)	0.17	0.003
	2-hour blood glucose (mmol/l)	−0.51 (−0.79 to 0.22)	0.0005	−1.88 (−3.33to −0.44)	0.01	−0.23 (−0.44 to −0.02)	0.03	<0.0001
	HbA1c (%)	−0.13 (−0.25to −0.001)	0.05	−0.54 (−0.97 to −0.11)	0.01	0.04 (−0.02 to 0.09)	0.18	<0.0001
	Albumin/creatinine ratio (mg/mmol)	−7.3% (−20.8 to 6.2)	0.29	−28.8% (−62.8 to 5.2)	0.10	1.0% (−12.6 to 14.5)	0.90	0.054
	Systolic blood pressure (mmHg)	0.22 (−1.85 to 2.29)	0.83	0.54 (−3.86 to 4.93)	0.81	0.13 (−2.20 to 2.47)	0.91	0.88

Models are adjusted for age, sex, diabetes and urinary albumin/creatinine ratio.

CKD-EPI, Chronic Kidney Disease Epidemiology Collaboration; eGFR, estimated glomerular filtration rate; MDRD, Modification of Diet in Renal Disease.

### Association between SNPs within MYH9 and CKD stage 3–5, and Albuminuria

In logistic regression analyses, none of the SNPs, single or in the presence of various combinations of quantitative and qualitative traits achieved a significant association with CKD stage 3–5 (eGFR<60 ml/min) based on either MDRD or CKD-EPI formulae (Table 4). However, effects were borderline with rs5756152, with a suggestion that allele G could be associated with about 34% to 48% lower risk of CKD, and consistently across definitions of CKD (Table 4). We also found no significant association between SNPs and proteinuria (ACR≥3.4 mg/mmol), irrespective of the level of adjustment for covariates (Table 5).

**Table 4:** Odd ration and 95% confidence intervals from logistic regression for the prediction of CKD stage 3–5.

Allele	Covariates	MDRD	p	CKD-EPI	p
rs5756152 G	None	0.66 (0.36–1.15)	0.17	0.61 (0.32–1.09)	0.12
	Age, sex	0.62 (0.32–1.14)	0.14	0.57 (0.27–1.08)	0.10
	Age, sex, diabetes	0.62 (0.31–1.13)	0.14	0.56 (0.27–1.07)	0.09
	Age, sex, ACR	0.56 (0.27–1.05)	0.09	0.52 (0.24–1.02)	0.07
	Age, sex, ACR, diabetes	0.56 (0.27–1.05)	0.09	0.52 (0.25–1.02)	0.07
	Age, sex, ACR, diabetes, diabetes*ACR	0.56 (0.27–1.05)	0.08	0.53 (0.25–1.04)	0.08
	Age, sex, ACR, diabetes, hypertension	0.57 (0.28–1.07)	0.10	0.54 (0.25–1.05)	0.09
	Age, sex, ACR, diabetes, HbA1c	0.55 (0.27–1.04)	0.08	0.52 (0.24–1.01)	0.07
	rs12107	0.57 (0.29–1.03)	0.08	0.53 (0.26–0.97)	0.05
rs12107 A	None	1.15 (0.77–1.70)	0.48	1.13 (0.75–1.68)	0.54
	Age, sex	1.23 (0.77–1.92)	0.38	1.23 (0.75–1.98)	0.40
	Age, sex, diabetes	1.24 (0.78–1.96)	0.36	1.24 (0.76–2.01)	0.38
	Age, sex, ACR	1.33 (0.83–2.08)	0.22	1.31 (0.80–2.12)	0.27
	Age, sex, ACR, diabetes	1.33 (0.84–2.08)	0.22	1.31 (0.80–2.12)	0.27
	Age, sex, ACR, diabetes, diabetes*ACR	1.33 (0.84–2.08)	0.22	1.32 (0.80–2.13)	0.27
	Age, sex, ACR, diabetes, hypertension	1.34 (0.84–1.12)	0.21	1.32 (0.80–2.15)	0.27
	Age, sex, ACR, diabetes, HbA1c	1.31 (0.83–2.06)	0.24	1.30 (0.79–2.10)	0.29
	rs5756152	1.21 (0.81–1.78)	0.35	1.19 (0.79–1.76)	0.39

ACR, urinary albumin/creatinine ratio; CKD-EPI, Chronic Kidney Disease Epidemiology Collaboration; eGFR, estimated glomerular filtration rate; MDRD, Modification of Diet in Renal Disease.

**Table 5:** Odd ratio and 95% confidence intervals from logistic regression for the prediction of proteinuria.

Covariates	rs5756152 G	P	rs12107 A	p
None	1.27 (0.80–1.95)	0.28	0.87 (0.59–1.26)	0.48
Age, sex	1.27 (0.80–1.95)	0.28	0.89 (0.60–1.30)	0.55
Age, sex, diabetes	1.28 (0.80–1.99)	0.28	0.89 (0.59–1.30)	0.56
Age, sex, CKD (MDRD), diabetes	1.30 (0.82–2.03)	0.25	0.89 (0.59–1.30)	0.55
Age, sex, CKD (CKD-EPI), diabetes	1.29 (0.80–2.01)	0.27	0.89 (0.59–1.30)	0.56
Age, sex, CKD (MDRD), diabetes, hypertension	1.33 (0.83–2.08)	0.22	0.91 (0.60–1.33)	0.63
rs12107	1.25 (0.77–1.98)	0.36	0.86 (0.58–1.25)	0.44

### Association between SNPs within MYH9 and other Traits

The associations of participants’ quantitative traits known to be related with CKD (including systolic blood pressure, fasting and 2-hour glucose and HbA1c) and the three SNPs were assessed in age, sex, ACR and diabetes adjusted regression models and after stratification by diabetes status (Table 3). rs12107 was associated with fasting-, 2-hour glucose and HbA1c in participants with diabetes (all p≤0.05) but only with 2-hour glucose in nondiabetics (p = 0.03), with always significant interactions by diabetes status (all interaction p≤0.003). rs5756152 was also associated with HbA1c in nondiabetics (both p≤0.03), but not in people with diabetes (both p≥0.09), with however significant heterogeneity by diabetes status (interaction p = 0.02). There was further suggestion that associations of rs5756152 with fasting and 2-hour glucose, although not significant, likely varied by diabetes status (both interaction p≤0.04). None of the SNPs was associated with systolic blood pressure (Table 3).

## Discussion

In this study we show that SNPs in the MYH9 gene are associated with renal phenotypes in a mixed ancestry population of South Africa with type 2 diabetes. This study contributes to existing knowledge in several key ways. Firstly, it has shown that polymorphisms within the MYH9 are associated with early changes in kidney function and urinary albumin excretion in a community based cohort as opposed to a patient population with clinically overt chronic kidney disease. Secondly, it has demonstrated that the associated MYH9 risk occurs at an early stage and is likely more pronounced in subjects with type 2 diabetes unlike previous studies which have shown a stronger association with nondiabetic end-stage renal disease. Thirdly, it identified rs5756152 to be the SNP with the strongest effect on renal phenotypes. Finally, the MYH9 SNPs also showed an association with CKD-related traits and those related to glucose control in particular such as blood glucose and glycated hemoglobin.

In mixed ancestry populations such as the African Americans and Hispanics, genome-wide studies have identified sequence variants in the MYH9 gene, that demonstrated a strong association with nondiabetic kidney disease [1], [2], [10], [11]. The mixed ancestry population of South Africa is also heterogeneous, with predominantly San-Khoi, African, European origin and a small proportion of Asian ancestry [21], but in contrast to these reports the MYH9 polymorphisms in this population group were strongly associated with renal traits in subjects with type 2 diabetes. Type 2 diabetes remains the strongest risk factor for renal diseases, but MYH9 polymorphisms are only weakly associated with diabetes related ESRD in both African and European Americans [12], [13]. Subjects with diabetes, particularly type 2 diabetes, in the presence or absence of diabetic nephropathy often have diabetes-unrelated kidney diseases [22], [23]. Our study is different from the previous ones as it demonstrates an early association between MYH9 polymorphisms and renal phenotypes and describes the effect of MYH9 polymorphisms in a community based cohort as opposed to clinically diagnosed ESRD. Our results suggest that subjects with MYH9 risk alleles have an amplified risk of CKD in the presence of type 2 diabetes, thus underscoring the importance to aggressively monitor subjects with type 2 diabetes for renal diseases.

Although the haplotypes, E-1, S-1 and L-1, confer all the excess burden of diabetes-unrelated ESRD in African Americans and Hispanics [1], [2], [10], [11], three SNPs, rs12107, rs5756152 and rs4821480, were found to be independently associated with diabetes-unrelated ESRD. After adjusting for E-1 and L-1 haplotype, rs5756152 and rs12107 exhibited independent evidence for association [11]. Similarly, Kao et al., showed significant associations between MYH9 SNPs that included rs12107, rs4821480 and rs5756152 and nondiabetic ESRD [2]. In our study, the strongest association with changes in eGFR was observed with rs5756152. Furthermore, rs5756152 G allele showed borderline associations that may account for more than 30% lower risk for CKD stages 3–5. Although rs4821480 was shown to be associated with CKD in individuals of European ancestry from the Framingham Heart Study [24], we could not confirm such an association. The lack of replication of these gene polymorphisms has been reported in other populations including those of African ancestry. For example, the L-1 SNPs were not significantly associated with non-diabetic ESRD in Hispanic Americans with varying degrees of Native American, African and European ancestries [10]. Similarly, in an HIV-infected African Ethiopian population the presence of a comparable frequency with African Americans of the E-1 haplotype was found in the absence of clinically apparent HIVAN [25].

Despite the identification of disease susceptible MYH9 SNPs, the disease causal mechanism remains largely unknown. The non-muscle myosin protein is abundantly expressed in the kidney, platelets and liver and in smaller amounts in the thymus, spleen, intestine and cochlea [3], [26], [27]. Within the kidney, it is expressed in the podocytes which are highly specialized cell, with the ability to ultrafilter blood and support glomerular capillary pressures [28], [29]. Although differentiation of podocytes has been associated with the up-regulation of actin-myosin molecules, MYH9 and MYH10 were not amongst those genes [29]. Herein, we did not find an association with blood pressure; instead we found that MYH9 polymorphisms affected blood glucose levels and glycated hemoglobin. The role of MYH9 in diabetes susceptibility has previously been proposed. Freedman et al [11] proposed that the association between MYH9 and diabetes associated ESRD could have resulted from MYH9 causing susceptibility to type 2 diabetes. Later the authors showed MYH9 polymorphisms specifically the E-1 haplotype to modulate the genetic effect of FERM domain-containing protein 3 (FRMD3) for diabetes susceptibility [30]. However, FRMD3 alleles were not associated with increased risk of type 2 diabetes in subjects carrying MYH9 E-1 risk alleles. Similarly, genome-wide linkage analysis has revealed genetic variants on chromosome 22, near the MYH9 and APOL1 to be linked to the pathogenesis of diabetes [31]. Taken together with the diabetes-renal disease risk, it is likely that MYH9 exerts this effect through disregulation of blood glucose metabolism, as such future work is warranted.

Our study also has important public health implications. This population is at an increased risk for CKD which is driven by a high prevalence of diabetes. An epidemic of diabetes in South Africa is magnified by the HIV/AIDS epidemic where the largest HIV infected population in the world resides [32], [33], [34], [35], [36]. Individuals with both HIV and diabetes are reported to be at significantly increased risk of progressive CKD compared with patients with only one commodity [37]. Furthermore, HIV associated nephropathy is strongly linked to MYH9 polymorphism in African Americans [38]. The aggregation of multiple risk factors on the development of CKD is of concern in this population. Therefore, our study and those previously reported emphasize the need to conduct prospective studies that can elucidate the value of genetic screening for CKD. Hopefully, such screening would lead to early differential diagnosis and treatment strategies that would delay CKD progression.

The limitations of this study include the design which is cross-sectional. The phenotypes were based on a single measurement. Further, the lack of data mapping the admixture ancestry markers limited our choice of SNPs. Although rs5756152 showed the strongest association with renal phenotypes, it is likely that other SNPs within the MYH9 may prove otherwise. Although, the MYH9 risk alleles are highly prevalent in populations with an African ancestry including the indigenous San and Bantu-South Africans [39] this is the first report confirming an association between MYH9 SNPs and renal phenotypes in a South African population.

In conclusion, rs5756152 is associated with early kidney function derangements in South African mixed ancestry subjects with type 2 diabetes whilst rs12107 is associated with glucose control. Our findings and other studies suggest that MYH9 may have a broader genetic risk effect on different types of kidney diseases. Further studies are needed to replicate our findings, and we recommend that these should include patients with diagnosed ESRD.
